# An Ethical Symposium, Being a Series of Papers Concerning Medical Ethics and Etiquette from the Liberal Standpoint

**Published:** 1883-12

**Authors:** 


**An Ethical Symposium, being a Series of Papers Concerning Medical Ethics and Etiquette from the Liberal Standpoint.** By ALFRED C. POST, WILLIAM S. ELY, S. OAKLEY VANDERPOOL, LEWIS S. PILCHER, THOMAS HUN, WILLIAM C. WEY, JOHN ORDRONAUX, DANIEL B. ST. JOHN ROOSA, C. R. AGNEW, ABRAHAM JACOBI and H. R. HOPKINS, with an appendix. New York: G. P. Putnam's Sons, 27 and 29 West Twenty-ninth St. 1883.

The preface states: "The object of this little book is to place before the reader the motives which induced a large number of medical men of the State of New York to dissent from some of the rules which have hitherto controlled and guided them in their intercourse with themselves and the public."

The work is published in the interests of the new code. The papers from the gentlemen whose names appear on the title page are other expositories of the principles on which the advocates of a more liberal policy on ethical questions base their arguments. We commend the work to our readers.

